# Immunoanalysis Methods for the Detection of Dioxins and Related Chemicals

**DOI:** 10.3390/s121216710

**Published:** 2012-12-05

**Authors:** Wenjing Tian, Heidi Qunhui Xie, Hualing Fu, Xinhui Pei, Bin Zhao

**Affiliations:** State Key Laboratory of Environmental Chemistry and Ecotoxicology, Research Center for Eco-Environmental Sciences, Chinese Academy of Sciences, Beijing 100085, China; E-Mails: tiantianstacy@163.com (W.T.); qhxie@rcees.ac.cn (H.Q.X.); hualing.flying@gmail.com (H.F.); xhpei@rcees.ac.cn (X.P.)

**Keywords:** ELISA, dioxins, Ah-I, multi-analyte immunoassay

## Abstract

With the development of biotechnology, approaches based on antibodies, such as enzyme-linked immunosorbent assay (ELISA), active aryl hydrocarbon immunoassay (Ah-I) and other multi-analyte immunoassays, have been utilized as alternatives to the conventional techniques based on gas chromatography and mass spectroscopy for the analysis of dioxin and dioxin-like compounds in environmental and biological samples. These screening methods have been verified as rapid, simple and cost-effective. This paper provides an overview on the development and application of antibody-based approaches, such as ELISA, Ah-I, and multi-analyte immunoassays, covering the sample extraction and cleanup, antigen design, antibody preparation and immunoanalysis. However, in order to meet the requirements for on-site fast detection and relative quantification of dioxins in the environment, further optimization is needed to make these immuno-analytical methods more sensitive and easy to use.

## 1. Introduction

Dioxin and dioxin-like compounds, including polychlorinated dibenzo-*p*-dioxins (PCDDs), polychlorinated dibenzofurans (PCDFs) and polychlorinated biphenyls (PCBs) are a group of chemicals classified as persistent organic pollutants (POPs). Most of them are man-made and released from waste incinerators [1,2] and many other industry processes, such as iron ore sintering, power plants and secondary aluminum smelters [3–5]. They are found ubiquitously in the environment, even in very remote locations, such as the Alaskan Arctic and Antarctica [6], and located in soils, sediments, air and water [7,8]. Because dioxins bioaccumulate in the food chain, they can have serious ecological effects and can impact human health, impairing fertility and development, attacking the immune system, posing neurological risks and causing cancer [9–11]. The multiple long-term health impacts of dioxins have been found in victims of exposure accidents. For example, in 1976, an industrial accident that occurred in Seveso (Italy) exposed a large population to substantial amounts of relatively pure 2,3,7,8-tetrachlorodibenzo-*p*-dioxin (TCDD). In early health investigations, chloracne was the only effect established with certainty. In long-term studies, however, an excess of diabetes and cancer cases were associated with dioxin exposure. Additionally, an excess mortality from cardiovascular and respiratory diseases was uncovered [12]. Furthermore, Koopman-Esseboom *et al.*[13] reported that elevated levels of dioxins and PCBs can alter the thyroid hormone status of pregnant women and their infants. Moreover, Weisglas-Kuperus *et al.*[14] found that Dutch preschool children who were exposed to PCBs and dioxins persisting into childhood might have a greater susceptibility to infectious diseases. In order to provide guidance on acceptable levels of exposure, the World Health Organization (WHO) has determined a tolerable daily intake of 1–4 pg·kg^−1^ body weight expressed as dioxin toxic equivalency (TEQ) [15]. TEQ represents total toxicity of a mixture of dioxins, each with its own toxic equivalency factor (TEF), expressing the toxicity relative to the most toxic dioxin congener TCDD. At the same time, scientists have been developing methods to analyze dioxins in environmental and biological samples, resulting in a variety of chemical analytical and bio-analytical detection methods for dioxins [16,17].

In the 1990s, the US Environmental Protection Agency (US EPA) published standard determination methods for dioxins: EPA Methods 1613 and 1618. These methods, viewed as the “gold standard”, employ high-resolution gas chromatography/mass spectrometry (HRGC/HRMS). They are considered to be the most reliable and sensitive methods for determining the congener-specific concentrations of dioxins. These methods were also adopted and standardized by other counties and organizations, such as Japan, WHO and European Union, but HRGC/HRMS has obvious disadvantages. First, this analytical method can only be performed by highly trained specialists. Second, it is very time-consuming. Usually, it takes three or four days to analyze one sample. Third, it requires extremely complicated and expensive pretreatments, costly instrumentation and high daily running cost to maintain a standardized laboratory. Between 1970 and 1993, it has been estimated that more than one billion US$ was spent on determining the toxicity of PCDD/PCDFs in samples in America [18]. Currently, it is estimated that one dioxin analysis costs in the range from $900 to $1,800. Moreover, there is an easily ignored problem on how to treat the large amount of solid matter and liquids polluted by dioxins during the analysis process. For example, analysis of one soil sample usually produces about four liters of liquid waste, and due to the high expense and complex detection procedures, it is hard to perform high throughput dioxin screening in large scale environmental surveillance using instrumental analysis. Therefore, it is urgent to develop more rapid, simple, cost-effective and sensitive methods. With the development of biotechnology, bioassays which could be rapid, simple and offer moderate sensitivity are being developed to detect dioxins [19]. Similar to instrumental analyses, TEQ of a particular class of dioxins can be analyzed and estimated according to the concentrations obtained by these methods (Table 1). The present bioassays of dioxins are divided into two categories: one is based on the aryl hydrocarbon receptor (AhR) signaling pathway activated by dioxins; the other, also called immunoassay, is based on antibodies. Because the former is correlated to the toxicological effects of dioxins in cells, making it physiologically relevant, total TEQ of dioxins in samples can be obtained. The latter, except for Ah-immunoassay (Ah-I) using antibodies to recognize AhR directly, use specific antibodies to identify dioxins, so it is not able to directly reflect the total toxicological and biological effects of mixtures of dioxins.

The detection methods for dioxin bioassays include quantitative polymerase chain reaction (Q-PCR) for CYP1A1 mRNA expression, the 7-ethoxyresorufin-O-deethylase (EROD) bioassay, the aryl hydrocarbon hydroxylase (AHH) bioassay, chemical-activated luciferase gene expression (CALUX), chemical-activated green fluorescent protein luciferase gene expression (CAFLUX), the gel retardation of AhR (GRAB) assay, cell proliferation-based assay, DNA binding assay, AhR ligand binding, enzyme-linked immunesorbent assay (ELISA), radioimmunoassay (RIA), dissociation enhanced lanthanide fluorescence immunoassay (DELFIA) and Ah-I (Table 1). Some of them have been standardized by different countries and organizations, such as the United States of America, Japan and European Union (Table 2). Each of these methods has its advantages and drawbacks. EROD is not useful for detecting trace dioxins due to its low sensitivity. Though CALUX is considered to be the best bio-analytical method for detecting TEQ concentrations of dioxins due to its high sensitivity and rapid detection [30], cell culture and other cellular biology facilities are necessary. CAFLUX does not require addition of an external substrate, unlike CALUX, but its background noise is high, which often prohibits its use. GRAB assay uses a ^32^P labeled DNA probe, requiring a radioactivity use license and facility and is mainly used in research. ELISA and RIA are based on the interaction of antigen and antibody therefore they have relative high specificity, and have the added benefit that cell culture is not required, unlike EROD or CALUX, but RIA requires antibodies labeled with ^125^I or ^32^P, again requiring a radioactivity use license, and is seldom employed. DELFIA is similar to the ELISA assay, but is more sensitive. A major drawback is its expense due to the requirement for special antigens labeled with lanthanide. An added concern is the risk of lanthanide ion contamination. Differing from the other antibody-based assays, the Ah-I assay, which is designed based on AhR signaling pathway, allows an estimation of the TEQ of mixtures of dioxins. All of these antibody-based bioassays require expensive antibodies. Despite this, antibody-based methods are the simplest, have moderate sensitivity, and are less expensive than HRGC/HRMS, leading to the development of commercial ELISA and Ah-I kits which have been utilized for dioxins screening [46–51]. At the same time, in order to improve detective sensitivity, several detection technologies can be combined as multi-analysis immunoassays, which will be described in detail later.

Developing detection methods suitable for screening dioxins on-site is of great importance for dealing with exposure accidents and daily surveillance. To fulfill the special requirement for on-site fast screening, the detection system must be simple, rapid, sensitivity and practical. Thus, bioassays based on antibodies may be the best suited for this purpose. Therefore, the present review focuses on several practical bio-analytical methods based on antibodies for dioxins such as ELISA, Ah-I and other multi-analysis immunoassays. First, the methodology and optimization of ELISA are summarized, in terms of the samples preparation, antigen design and antibody productions. In the following part, the Ah-I bioassay and its applications are introduced. Finally multi-analyte immunoassays are described. Other screening methods based on antibody-antigen reactions have been highlighted in numerous excellent reviews [19,52,53].

## 2. Detection Methods

### 2.1. ELISA

**Figure 1.**
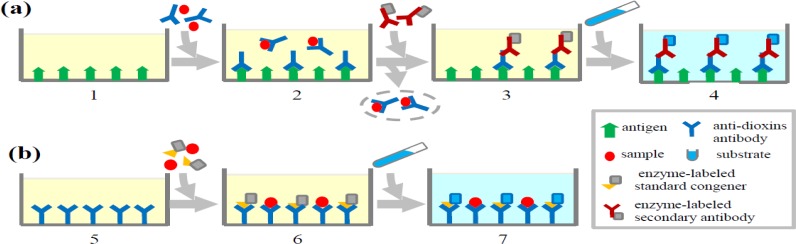
Schematic diagram for experimental procedure of indirect and direct competitive ELISA for detecting dioxins. (**a**) For an indirect competitive ELISA assay, a microplate is coated with appropriate antigens (1). Samples and anti-dioxin antibodies (Abs) are added in to the microplate. Antigens compete with dioxins in the test sample for binding to the Ab (2). After incubation, soluble molecules are washed away. Secondary antibody labeled with enzyme is added and reacts with the immobilized anti-dioxin Abs (3). Substrates are added to detect the immobilized Ab (4). (**b**) For a competitive ELISA assay, a microplate is coated with anti-dioxin Ab (5). After incubation, soluble Abs are removed by washing. Samples and enzyme-labeled standard congener are added which compete for binding to the anti-dioxin Abs. After incubation, soluble molecules are washed away (6). Substrates are added to detect immobilized standard congener (7).

ELISA is based on the specific reaction between dioxins and their antibodies. Currently the predominant ELISA systems used for detecting dioxins are direct competitive and indirect competitive ELISAs which are illustrated in Figure 1. The basic principle of competitive ELISA is that dioxins in environmental and biological samples compete with standard congener conjugated with enzyme for binding to antibody against a certain class of dioxin congener immobilized on a microplate. The substrate reacts with enzyme-linked standard congener and gives absorbance which reflects the amount of conjugated standard congener binding to the antibody on the plate. The absorbance is inversely in proportion to the amount of dioxins present in the tested samples. The concentrations of dioxins in the samples are calculated by comparison with a standard curve generated by addition of known amounts of standard dioxin.

Indirect competitive ELISA is based on the competition between dioxins in samples and antigens immobilized on the microplates for binding to antibodies that recognize a certain class of dioxin congener. After washing to remove soluble antigen-antibody complexes, the antibody bound to the antigen on the plate is then incubated with secondary antibody conjugated to an enzyme which can subsequently react with substrate to give an absorbance. Like the direct competitive ELISA, dioxin levels in the samples can be evaluated by comparison with a standard curve. Although ELISA is a simple assay for determination of dioxins, its respective lack of sensitivity and specificity compared to CALUX and instrumental analyses limits its applications. To make improvements, several aspects should be considered, including the optimization of sample preparation and improvement of the specificity of antibodies against dioxins. The latter is related to the haptens chosen for antigen design and the technology for generating antibodies.

#### 2.1.1. Sample Preparation

Sample preparation is important for concentrating the dioxins in the mixture, removing potential interfering ingredients and improving the sensitivity of the assay. HRGC/HRMS requires exhaustive extraction and expensive cleanup for sample preparation which is expensive and time-consuming, while the sample preparations for ELISA assays are usually fast, simple and cost-effective. Moreover, new methods for sample extraction and cleanup are being developed for better determination of dioxins using ELISAs.

##### Sample Extraction

Samples from different sources are accompanied by distinct complex matrices, requiring different extraction methods. In the early days, soil and sediment samples were easily extracted by shaking with methanol or/and Soxhlet [47,54,55] or using an ultrasonic process [56], but these methods did not result in a complete extraction, leading to compound loss or the need for further cleanup. In order to solve these problems, several alternative extraction technologies were introduced, which resulted in time and reagent savings. Microwave energy has been used to extract organic compounds from contaminated soils for many years. Microwave-Assisted Extraction (MAE) is fast (*i.e.*, the sample is statically extracted within 10–15 min) and solvent-saving (*i.e.*, 30 mL per sample as compared to 300 mL for instrumental analysis). Lopez-Avila *et al.*[57] detected PCBs in soils and sediments with this method. Five gram soil samples were subjected to extractions with only 30 mL hexane-acetone (1:1), and the extraction was performed for only 10 min. The subsequent analyses showed a good match between MAE-ELISA and MAE-GC/ECD results with a correlation coefficient over 0.97.

Supercritical fluid extraction (SFE) is an alternative efficient extraction for some special samples such as oily soils. It is a technique combining the liquid-like solvating and gas-like transport properties of supercritical fluids. Because pure carbon dioxide (CO_2_) is used as solvent, extractions are clean enough for direct reconstitution in a solvent compatible with ELISA [58–61]. For example, Johnson *et al.*[51] studied the extraction of PCBs from oily soil using both a methanol shake extraction and a methanolic Soxhlet extraction, but found that the ELISA results disagreed with the GC confirmatory data. However, SFE with high pressure/temperature regime coupled to the same ELISA, resulted in a good agreement between the results obtained by GC and by ELISA. Additionally, Bøwadt *et al.*[62] also examined PCBs in soils using a SFE method without the need for cleanup steps after extraction. The following ELISA performed under laboratory conditions gave reasonable agreement with GC/ECD for determining total PCBs.

Accelerated solvent extraction (ASE) uses a conventional solvent to extract samples, but at elevated pressure and temperature. Like MAE, it is also fast and consumes only small amounts of solvent. Soil samples heavily contaminated by PCDD/Fs from small-scale industrial waste combustion sites in Uruguay, wood treatment sites in Sweden and a Swedish chloralkali site were put into 33 mL stainless steel extraction cells filled with solvents. The toluene fractions, containing the PCDD/Fs, were obtained in 43 min, which is considerably faster than using Soxhlet extraction with toluene (15 h). The ELISA results were strongly correlated with HRGC/HRMS results (correlation coefficient, 0.90) [63]. Schrock *et al.*[64] also used ASE to extract sediments and soils contaminated with PCDD/Fs. The extractions were carried out at 13.8 MPa (2,000 psi), 125 °C and three static cycles of 10 min each. There was also a high correlation between ELISA and GC results, with a coefficient at 0.99.

Selective pressurized liquid extraction (SPLE) is another relatively new technique. In this method, sulphuric acid-impregnated silica was used for the lipid free extraction of compounds by pressurized liquid extraction. Similar to SFE, it also reduced the need for post-extraction cleanup. For instance, Chuang *et al.*[65] adopted this method to extract sediments and soils contaminated by PCDD/Fs. The ELISA results demonstrated that this method was an effective extraction method for a wide range of PCDD/F concentrations in sediments and soils without the need for further cleanup.

For contaminated oil samples, the extraction is usually less complicated. For example, Samara *et al.*[66] studied industrial source oil samples contaminated by PCDD/Fs, which were cleaned up by means of Soxhlet extraction with methylene chloride (3.5 h) under restricted exposure to light. In addition, Glass *et al.*[67] reported a less complicated simple two-step liquid-liquid extraction which was used to detect PCBs in transformer oil, resulting in no false negatives at a 1.4 ppm nominal cutoff. Moreover, using a simple oxidation of the sample extract (including 1 mL acetonitrile, 0.5 mL isooctane, 1 mL H_2_SO_4_ and 8 mL “Quenching Buffer”) to analyze PCBs in waste oil, ELISA results were highly correlated with the results obtained by instrumental analysis [68].

With biological samples, different extraction methods are available for obtaining reliable ELISA results. Wiberg *et al.*[69] used SPLE to treat food and feed samples contaminated by PCDD/Fs and dioxin-like PCBs. The ELISA results showed good agreement with HRGC/HRMS. Similar to environmental samples, SPLE also reduced time and solvent consumption compared to instrumental methods. In addition, different sample preparation methods were used for fish. Fish samples were mixed with sodium sulfate, extracted with methylene chloride and fortified with the surrogate congener [70]. Tsutsumi *et al.*[71] reported another method for sample preparation of retail fish tissue, in which the homogenized fish sample was first subjected to alkali digestion, and then, with addition of methanol, it was extracted with hexane and finally washed with aqueous NaCl. The ELISA results correlated well with TEQ concentrations for dioxin-like PCBs obtained by HRGC/HRMS (r = 0.92, n = 26). Invertebrate animals such as mussels exposed to PCBs were also studied by Fillmann *et al.*[72]. The samples were Soxhlet extracted in hexane-dichloromethane (1:1), and the extracts were then concentrated to a few mL using rotary evaporation followed by pure nitrogen “blow down” The ELISA results indicated that it was a rapid and efficient extraction for detecting total PCBs in mussels.

##### Cleanup

In order to make the extracted sample compatible with ELISA assays, samples must be re-dissolved in dimethyl sulfoxide (DMSO) before assay. In addition, cleanup is usually needed after extraction to eliminate interfering ingredients. Several general chemical methods have been used for the cleanup. Tsutsumi *et al.*[73] cleaned up sample extracts from retail fish contaminated by dioxin-like PCBs by passing them through a multi-layer silica gel column and an alumina column, resulting in eluents suitable for ELISA assays. Deng *et al.*[55] cleaned soil and sediment samples on a column containing activated Florisil [74]. Nichkova *et al.*[56] utilized a multilayered silica gel column and an activated carbon column to clean up extracted sediment samples contaminated by PCDD/Fs. In addition, Van Emon *et al.*[75] used the same method to refine dioxins-contaminated sediment and soil sample extracts, following the EU standard methods. For the cleanup of samples extracted from oils, KOH-ethanol/sulphuric acid is suitable for oil samples containing > 35 μg·mL^−1^ PCB [76].

For biological samples containing extremely low concentrations of dioxins, such as human milk, Sugawara *et al.*[77] reported that routine purification with nonpolar solvents was incompatible with ELISA assays. They suggested extracting human milk samples by employing an alkali decomposition step, then passing the samples through a three-layer column, and finally evaporating the eluent with nitrogen before re-dissolving in MeOH-DMSO (1:1) with 100 ppm Triton X-100. A fairly good correlation between ELISA results and GC/MS results was obtained with a coefficient of 0.92.

As an alternative to traditional chemical cleanup methods, immunoaffinity purification (IAP) is a powerful biochemical technique for a single-step separation and purification of individual compounds or classes of compounds from liquid matrices [78–80]. IAP is based on the highly specific interaction of an antigen in the samples with its antibody immobilized on a solid support matrix. It was originally applied to pharmaceutical and biomedical trace analysis and in recent decades for analysis of environmental contaminants and pesticide residues [81,82]. Altstein *et al.*[83] first coupled ELISA with the sol-gel-based IAP method for cleanup of soil and sediment samples for determination of PCB 126, demonstrating that this method was very efficient in purifying PCB 126 and eliminating matrix interferences. Shelver *et al.*[84] also used an immunoaffinity column for cleanup of TCDD containing serum samples. The binding efficiencies of a spiked serum sample combined with the immunoaffinity column were over 90%.

#### 2.1.2. Immunogen Design for PCDD/Fs and PCBs

The specificity and selectivity of ELISAs mainly depend upon the antibodies used. Many studies show that the design of the immunogens determines the features of the resulting antibodies [85–88]. Since small molecules seldom stimulate an immune response, it is necessary to design a hapten which can be covalently combined with a carrier protein to make a complex for immunization. Usually two aspects of a hapten are related to the immunogenicity: chemical structure and functional group(s). The chemical structure is sometimes the key to produce valuable antibody. The functional groups (e.g., NH_2_, COOH) help to combine chemical structures with carrier proteins by covalent coupling.

A lot of effort has been made to detect various PCB congeners. Chiu *et al.*[89] designed a hapten (6-[(3,3′,4′-trichlorobiphenyl-4-yl)oxy]hexanoic acid) coupled to bovine serum albumin (BSA) by which an appropriate monoclonal antibody for sensitive detection of PCB congeners or mixtures was obtained. The ELISA using the antibody was highly selective for PCB 77 and PCB 126, with 50% inhibition concentration (IC_50_) values of 0.9 and 1.2 ng·mL^−1^, respectively. Altstein *et al.*[83] used a 3,3′,4,4′-trichloro-(3-thiobiphenyl) hapten linked to keyhole limpet hemocyanin by means of 6-bromohexanoic acid to obtain a goat 2504 anti-PCB polyclonal antibody. The goat anti-PCB IgGs exhibited the highest affinity and lowest detection limit for PCB 77 and did not cross react with any other tested PCBs, Aroclors or furans in any of the ELISA formats. Fránek *et al.*[90] synthesized PCB 126-(CH_2_)_3_-hapten mimic-BSA and PCB 77-CH_2_-hapten mimic-peroxidase. The antibody was highly specific for non-*ortho*-substituted (coplanar) congeners, with low cross-reactivity values for PCB 189 and PCB 77 (1.3 or 1.0%, respectively) and did not recognize non-coplanar PCB congeners or organochlorine compounds. Fránek *et al.*[91] also synthesized a 4,4′-dichlorobiphenyl- and 3′,4,4′,5-tetrachlorobiphenyl-2-azo-BSA immunogen and obtained a sheep polyclonal antibody. Direct competitive ELISA results showed IC_50_ values for the commercial PCB mixture, Delor 103,104,105 and 106 (corresponding to Aroclor 1242, 1248, 1254 and 1260), were about 200, 250, 600 and 900 pg·L^−1^, respectively, and the cross-reactivity to Delor 103, 104, 105 and 106 was 100, 79.6, 31.7 and 13.5%, respectively. Furthermore, a 3,3′,5,5′-tetrachlorobiphenoxybutyric acid (PCBH)-keyhole limpet hemocyanin conjugate was also prepared by Inui *et al.*[92]. The resulting monoclonal antibodies, 0217 and 4444, were highly selective for 3,3′,5,5′-tetrachlorobiphenyl (PCB 80) in ELISA assays with IC_50_ values of 2.6 and 0.46 ng·mL^−1^, respectively. Moreover, Shan *et al.*[74] designed a hapten (7,8-dichlorobenzo[5,6][1,4]dioxino[2,3-b]pyridine-3-carboxylic acid) conjugate with BSA and obtained a sensitive polyclonal antibody for screening TCDD from soil and biota samples.

#### 2.1.3. Development of Antibodies for PCDD/Fs and PCBs

Among various bioassays for dioxin determination, one of the outstanding advantages of immunoassays is their specificity, which depends on the characteristics of the antibodies recognizing target analytes (antigens). To date there are several successful antibodies being used for ELISA assays of PCDD/Fs in environmental samples. Early work in this area has been summarized by Sherry et al. [93] and Harrison et al. [94]. So far there are three main kinds of antibodies used in the ELISA: polyclonal antibodies, monoclonal antibodies and recombinant antibodies. Polyclonal antibodies are widely used and easy to be obtained and purified from serum. For example, Mapes et al. [54] utilized New Zealand white rabbit to raise polyclonal antibodies against PCBs in the development of a fast on-site immunoassay. By using this assay PCBs in soil could be detected at concentrations as low as 5 ppm. The results of cross-reactivity studies showed that the assay was highly specific. Chuang et al. [50] also used pooled rabbit anti-PCB antiserum (AC-3) to determine PCBs in soil, and obtained a correlation coefficient between GC/ECD and ELISA of 0.91 for 41 soil samples. In addition, a sheep polyclonal antibody was developed, which had no cross-reaction with 19 other structurally related compounds, and only 5% cross-reactivity with dichlorodiphenyledichloroethylene (DDE) [91]. Deng et al. [55] also applied the sheep antiserum raised against 4,4′-dichlorobiphenyl- thyroglobulin immunogen to successfully evaluate eight PCB-contaminated soil samples. Besides polyclonal antibodies used for detection of PCB congeners, Carlson et al. [95] used a novel anti-PCDD/Fs polyclonal antiserum to measure TEQ of PCDD/Fs from soil and fly ash samples.

It is generally accepted that polyclonal antibodies are not absolutely specific and often exhibit cross-reactivity with other molecules. In the study of Harrison *et al.*[96], a competitive ELISA assay based on a mouse monoclonal antibody, which was specific for TCDD and related congeners, was documented. The sensitivities were 100 pg/tube or 25 pg/well of TCDD. Moreover, Tsutsumi *et al.*[71] used a monoclonal antibody, which was highly specific for 2,3′,4,4′,5-pentachlorobiphenyl (PCB 118), to determine contamination of retail fish. The ELISA results for the fish samples correlated well with the TEQ concentrations of dioxin-like PCBs obtained by GC/MS (r = 0.92, n = 26).

In most cases, cross reactivity is viewed as undesirable, and minimizing it is a common goal. However, since a mixture of different classes of dioxin congeners is always present in environmental samples, a general environmental screening of dioxins requires detection of certain classes of compounds rather than individual congeners. Therefore cross reactivity among different classes of the dioxin congeners, rather than individual congeners, is sometimes preferably considered in the development of immunoassays for dioxin screening. A new strategy to raise suitable antibodies has been put forward based on the notion of “generic” antibodies with relatively broad cross reactivity to targeted compounds [97]. This approach involves either screening of antibodies raised against a family of target compounds or generation of specially designed analogues, often based on a fragment of the target compounds aiming at presenting the pivotal features of the entire class [98,99]. Some researchers have described the use of mixed antibodies to increase the working range of an immunoassay for detecting herbicides [100,101]. A similar idea has been used to develop immunoassays for dioxins. For instance, Glass *et al.*[102] first described an immunoassay for PCBs in which two different monoclonal antibodies. As a supplement, cross reactivity was used to broaden the assay reactivity. Antibodies that individually exhibit 20 or 50-fold differences in response between Kanechlor 300 and Kanechlor 600 can be mixed so that the overall difference in response to those compounds was reduced to three fold. The reported cross reactivity of a PCB immunoassay was increased, resulting in an assay system which was better able to detect total PCBs.

Polyclonal and monoclonal antibodies dominated the dioxin screening field in the past and will continue to do so in the near future. But recent years, some researchers have begun to study recombinant monoclonal antibodies. The technology for producing recombinant monoclonal antibodies is not novel, originating in the medical field for generating humanized antibodies [103–105]. Before being used in dioxin mornitoring, it was introduced for screening pesticide [106–108]. Recombinant monoclonal antibodies are created from cDNAs encoding available monoclonal antibodies or taken from human cells, then cloning them into expression vectors for production in suitable organisms such as *Escherichia coli* or yeast. The preparation is highlighted in an excellent review [109]. Recombinant monoclonal antibodies have some advantages over conventional polyclonal antibodies and monoclonal antibodies. They are stable and have high affinity and low production cost. However, the procedure for their production is very complicated and requires highly specialized bioengineering techniques. The main advantage of recombinant monoclonal antibodies for ELISA analysis is its high tolerance towards solvents such as methanol and DMSO, alleviating the usual problem of having to greatly dilute the samples before assay [72,110]. Although some studies reported that ELISA system for coplanar PCBs were tolerant to organic solvents such as 5% of methanol and 1.3% of DMSO [89,90], these tolerances still decreased sensitivity for PCBs with standard antibodies.

### 2.2. Ah-I

**Figure 2.**
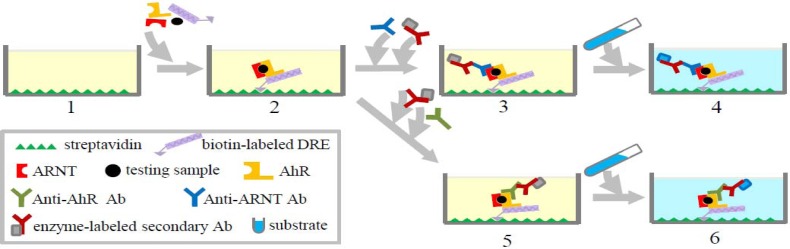
Schematic diagram for experimental procedures of Ah-I for detecting dioxins. Microplates are coated with streptavidin (1). Cell lysate containing AhR and ARNT, testing samples containing dioxins, and biotin-labeled DNA fragments containing DRE consensus sequences are added into the microplate. Biotin-labeled DRE binds to streptavidin. AhR activated by dioxins combines with ARNT to form a complex, which is immobilized by binding to DRE (2). Anti-ARNT antibody (3) or anti-AhR antibody (5) is added to recognize the complex. Secondary antibodies and substrates are added for detection (3, 4, 5, 6).

Ah-I is another common method to detect total toxicity potential of dioxins based on the ability of these compounds to bind to and activate AhR followed by downstream signaling. For determining TEQ concentrations of dioxins, reporter gene assays, such as CALUX, are currently regarded as the best method, especially for food and feed [30]. However, this still has some drawbacks, including the need for cell culture, which requires skilled personnel and elaborate equipment, and licensing. Ah-I, which is an ELISA-based AhR binding assay, is a simpler alternative to obtain TEQ directly that does not require cell culture. It is a hybrid of an immunoassay and an *in vitro* AhR-binding assay. The principle of this method is as follows: activation of AhR by dioxin or dioxin-like compounds leads to conformational changes, allowing the formation of a heterodimer with ARNT which then binds to dioxin receptor elements (DREs) in DNA. This AhR:ARNT heterodimer can be detected by an immunoassay-based color reaction using an enzyme-conjugated antibody that binds to the ARNT or AhR. The assay, which converts into TCDD equivalents using a TCDD standard curve, can detect the total TEQ concentrations of the dioxin-like compounds. The detailed procedure of this method is shown in Figure 2. The sensitivity and specificity of Ah-I are determined by sample preparation methods as well as by the features of the antibody used in the assay.

Like all of the bioassays based on antibodies, Ah-I also requires expensive antibodies, limiting its applications. One of the main advantages of this method is that it is less likely to produce false-negative results than cell-based assays, such as the CALUX, because Ah-I is based on very specific antigen-antibody recognition. For example, PCB 118, which is a good example of mono-*ortho* PCBs in certain kind of fish, was reported to be relatively less responsive in the CALUX assay [111–113], which may lead to underestimation [114]. However, according to studies of Tsutumi *et al.*[45], amounts of PCB 118 detected by Ah-I were consistent with the results of GC/MS analysis. On the other hand, sample cytotoxicity resulting from toxic matrices or solutions, may generate false negative results in CALUX, but in are less likely to interfere with the cell-free Ah-I assay. So far the detection limit of Ah-I assay for PCBs was 10 fmol.

### 2.3. Multi-Analyte Immunoassay

ELISAs have been generally considered to be rapid, simple and low-cost analytical procedures and used to detect dioxins for years. However, the sensitivity of these methods is limited. Using modified extraction and cleanup methods, the limit of detection was 28 ± 6 pg TMDD·mL^−1^ DMSO [2,3,7-trichloro-8-methyl-dibenzo-*p*-dioxin (TMDD)] [63]. For CALUX, the limit of detection was 50 fg of TCDD [112]. Therefore it is difficult to use ELISA assays for the evaluation of biological samples containing dioxins at extremely low concentrations, such as in human milk and blood. Since improvements in the ELISA assay itself may be not enough to make the assay suitable to detect trace amount of dioxins, novel detection systems consisting of other bio-analytical methods and ELISA have been developed for the purpose.

Real-time immuno-polymerase chain reaction (Rt-IPCR) is a versatile and robust technique whereby the exponential amplification ability of PCR is coupled to the detection of proteins by antibodies in an ELISA format. Briefly the antigen is coated on PCR tubes, and then anti-dioxin antibodies and samples containing dioxins are added. The antigen competes with dioxins in the sample for binding to the antibodies. Biotinylated secondary antibody is added to bind to anti-dioxin antibodies immobilized on the tube. After addition of avidin and biotinylated DNA tag, a complex is formed between avidin and the biotinylated secondary antibody and DNA. The DNA tag is then amplified and quantified by Rt-PCR. Rt-IPCR has been widely used in clinical diagnosis and biochemical analysis for detection of pathological proteins and various viral antigens [115–117]. Recently, it has been introduced to detect environmental contaminants, such as dioxin. For example, Chen *et al.*[118] first developed a fluorescent quantitative IPCR and assessed its potential for the detection of antibodies recognizing PCBs in soil samples, in which the PCB concentrations were as low as 10 fg·mL^−1^. Recovery rates were 95.0–105.0% and the Rt-IPCR results correlated well with the concentration of PCBs obtained by GC/MS (r = 0.99, n = 6). These data indicate that this highly specific, sensitive, and robust assay could serve as a potential tool for detecting PCB compounds in the environment. However, there are several disadvantages of Rt-IPCR that needed to be mentioned. Above all, it is hard to avoid laborious preparation to stably couple the DNA tag to the antibody. In addition incubation and washing steps are laborious compared to other competitive immunoassays. Finally, the sensitivity of Rt-IPCR is limited by antibody-antigen interaction.

Although ELISAs are rapid, these methods still require an hour at least for a single measurement and need enzyme-labeled reagents. Surface Plasmon Resonance (SPR)-based biosensor immunoassays (BIAs) using Biacore instruments can eliminate the need for enzyme-labeled reagents and are mostly highly automated. BIA is mainly used to detect the residues of veterinary drugs in foodstuffs, but recently it was introduced to screen dioxins. Using this method, Shimomura *et al.*[119] detected TCDD and PCB with respective detection limits of 0.1 and 2.5 ng·mL^−1^, requiring 15 min for a single sample measurement. The first food monitoring program using BIA was reported by Tsutsumi *et al.*[70]. In this study, an SPR-based BIA was developed using a monoclonal antibody that is specific for PCB 118, by which TEQ concentrations of dioxin-like PCBs in retail fish was obtained. The quantitative limit of the assay was 1 ng PCB 118 per gram of test samples and the assay results correlated well with GC/MS (r = 0.89). Thus, the BIA will be useful for the preliminary screening of large numbers of fish samples before GC/MS. However, BIA is not appropriate for detecting high concentrations of dioxins as it takes too long to complete dissociation of magnetic particles from the gold film which acts as a SPR tag. Another drawback is that this method requires expensive equipment and supplies.

## 3. Conclusions

Compared to GC/MS detection of dioxins, bioassays based on antibodies such as ELISA and Ah-I are more rapid, time-saving, cost-effective and practical. The cost-effective features of the bioassays are not only due to the relative low cost of the establishment of the detection system, but also because of the low cost for the maintenance of the instruments and sample preparation and detection. However, their drawbacks in sensitivity and specificity preclude them as a replacement for the instrumental method. In order to improve sensitivity and specificity, efforts have been made to optimize the sample preparation, hapten design and development of the antibodies. Combinations of other techniques with the antibody-based bioassays have been proposed to improve the performance in screening dioxins. For sample extractions, MAE, ASE, SPE and SPLE methods have been proposed, among which SPE and SPLE can save the cleanup step for the extracted samples; MAE and ASE are fast and use little solvent. For the cleanup of the extracted samples, multilayered columns and an activated carbon or IP are easy and effective methods. In order to obtain suitable antibodies, a well-designed hapten is essential. In general, polyclonal antibodies are easier to obtain, but monoclonal antibodies are more specific. ELISA assays using a mixture of various monoclonal antibodies can be employed to screen different classes of dioxin congeners. In addition, recombinant monoclonal antibodies can be employed in ELISA to improve the feasibility of the assay. Compared to single-analytic antibody-based analysis, multi-analytic analyses are able to not only further improve the sensitivity and specificity of the detections, such as Rt-IPCR; but also make the assays more rapid and automatic, such as BIAs. With the cell-free, simple and rapid features, these antibody-based bioassays could be feasible for direct on-site screening for dioxins. To make good use of all the optimizations, for example, dioxins in soils and sediments could be screened on-site by using MAE extraction and IA cleanup to prepare samples, followed by Ah-I to estimate the total TEQ. If the TEQ is relative high, ELISA can be adopted to determine different classes of the dioxins. If the TEQ is relative low, it is better to adopt Rt-IPCR. Alternative sample preparations using SPE or SPLE combined with BIAs can be used to screen a sufficient number of samples in a short time.

Ah-I can be used for specific applications. Although Ah-I can provide TEQ directly in a cell-free system, it has an obvious drawback in its specificity. Recently, more and more AhR ligands have been found which have diverse structures. The emerging evidence suggests that various ligands of AhR may bind to AhR in different ways [120] and these new findings suggest a possibility that once AhR is activated by different ligands, it may undergo distinct conformational changes. If there is an antibody specifically recognizing a certain conformational change induced by certain ligand, it would be possible to increase the specificity of Ah-I. The question is how to obtain such antibodies. So far antibodies used in Ah-I assays are mainly raised against short peptides derived from the AhR protein. Using this method to generate antibodies will result in a smaller probability of obtaining antibodies against special conformational changes compared to using full-length AhR as an antigen. With the further optimization, it may be possible to make these immune-analysis methods more sensitive and feasible to meet the requirements for on-site fast detection and relative quantification of dioxins in the environment.

## Figures and Tables

**Table 1.** Principle of biological detection methods for dioxins.

**Method**	**Principle**	**Reference**
(I) AhR signal pathway	When cell lines are exposed to dioxins, cytoplasmic AhR is activated, enters the nucleus and makes a complex with ARNT (aryl hydrocarbon receptor nuclear translocator). The complex binds to genomic DRE elements inducing the expression of CYP1A1 gene transcripts, measured by one of the assays below (a–e).	
(a) Q-PCR	By detecting the increase in CYP1A1 mRNA levels gene, the TEQ of dioxins is deduced, compared with a TCDD standard.	[20,21]
(b) AHH/EROD	Enzymatic activity of CYP1A1 is determined using the substrates 7-ethoxyresorufin or benzo[a]pyrene. The TEQ of dioxins is deduced, compared to a TCDD standard.	[22–25]
(c) CALUX	Recombinant cell lines, stably transfected with dioxin responsive firefly luciferase reporter genes, are exposed to dioxin-containing samples. When dioxins activate AhR, the AhR-ARNT (aryl hydrocarbon receptor nuclear translocator) complex will be formed which binds to DREs and induces expression of the luciferase reporter gene. Luciferase is easily assayed in a luminometer after addition of luciferin substrate.	[26–29]
(d) CAFLUX	The assay is similar to CALUX except enhanced green fluorescent protein is used as the reporter gene. The TEQ of dioxins is determined by measuring the intensity of the fluorescence emitted by the reporter.	[30–32]
(e) GRAB	Samples are incubated with hepatic cytosol extracts containing AhR and ARNT. Dioxins in the samples induce the formation of activated AhR/ARNT complexes. When the ^32^P isotope labeled DNA probes containing DRE consensus sequence bind with these complexes, the migration rate of the DNA probes in polyacrylamide gels will be slowed down compared to unbound probe.	[33,34]
(II) Cell proliferation-based assay	Dioxins are potent inducers of chloracne in humans, which is characterized by hyperkeratinization. By detecting the abnormal terminal differentiation of skin cells exposed to the sample, the TEQ of dioxins can be obtained compared with a TCDD standard.	[35,36]
(III) AhR ligand binding	Dioxins in samples compete with isotope labeled TCDD for binding to AhR. Therefore, the concentration of the dioxins in the sample is proportional to the decrease of radioactivity of AhR.	[37]
(IV) Immunoassay (a) ELISA	This assay is based on binding of dioxin to anti-dioxins antibodies. Dioxins in the samples compete with standard congener immobilized on the plate for binding to anti-dioxin antibodies. After washing away free antibody-antigen complexes, the antibodies bound to immobilized TCDD are detected by binding of secondary antibodies conjugated to an enzyme, followed by enzyme assays using a substrate which is easily measured. Alternatively, dioxins in the sample compete with standard congeners conjugated to an enzyme for binding to anti-dioxin antibodies immobilized on the plate followed by washing and enzyme assays. By using a standard curve generated by known concentrations of dioxin standards, the TEQ of dioxins can be deduced.	[38,39]
(b) RIA	Radiolabelled standard antigens (*Ag) and unlabeled sample antigens (Ag) compete with inadequate specific antibodies (Ab) and form *Ag-Ab or Ag-Ab complexes. When the binding reaction to achieve dynamic balance, if Ag quantity increase, Ag-Ab quantity increase, while *Ag-Ab relatively reduce and free Ag increase. Namely the mass of Ag is inversely proportional to the mass of radiolabelled complexes. Complexes and free antigens are effectively separated, and then measure the radioactivity to get the content of samples antigen.	[40,41]
(c) DEFIA	Similar to competitive ELISA, except using Eu to label the antigen or antibody.	[42,43]
(d) Ah-I	According to AhR signaling pathway, the amount of AhR:ARNT heterodimer is correlated to the concentration of the dioxins. First to immobilize AhR:ARNT complex by DNA fragments containing DRE consensus sequences which are linked on the microplates. Then the immobilized AhR:ARNT complex is detected by using anti-AhR or anti-ARNT antibody.	[44,45]

**Table 2.** List of standard bioassays for detection of dioxins.

**Standard number**	**Method**	**Sample**	**Detection chemicals**
US EPA 4020	Immunoassay	Soils, non-aqueous waste liquids	PCBs
US EPA 4025	Enzyme immunoassay	Soils	PCDD/Fs
US EPA 4425	A reporter gene on a human cell line	Soils, sediments tissues, water	PCBs, PCDD/Fs
US EPA 4430	AhR-PCR assay	Soils, sediments	PCDD/Fs
US EPA 4435	CALUX bioassay	Soils, sediments	Dioxin-like compounds
USEPA/600/R-01/052	DELFIA™	Soils, solvent extract	PCBs
2002/69/EC	Cell-based bioassays	Foodstuffs	PCDD/Fs, Dioxin-like PCBs
